# LC-MS/MS Quantification and Comparative Profiling of Stratum Corneum Ceramides in Human Normal and Dry Skin Subtypes

**DOI:** 10.3390/metabo16040260

**Published:** 2026-04-13

**Authors:** Agui Xie, Yue Zhao, Yu Zhao, Xiao Zhao, Xiaoge Zhu, Jia Wang

**Affiliations:** 1 Corporate Research and Development Center, Pientzehuang (Shanghai) Biotech R&D Co., Ltd., Shanghai 200030, China; xieagui@pzhrd.com (A.X.); zhaoyue@pzhrd.com (Y.Z.); zhaoyu@pzhrd.com (Y.Z.); zhaoxiao@pzhrd.com (X.Z.); 2 Department of Epidemiology and Biostatistics, Zhengzhou University, 100 Kexue Avenue, Zhengzhou 450001, China

**Keywords:** ceramides quantification, sensitive dry skin, LC-MS/MS, skin barrier function, lipidomics

## Abstract

**Background**: Ceramide (Cer) dysregulation in content and composition is linked to various skin conditions, particularly sensitive and dry skin. Existing ceramide quantification methods often lack efficiency, sensitivity, or comprehensive analytical capabilities. This study aimed to adopt an optimized LC-MS/MS platform to ensure the acquisition of reliable and accurate ceramide quantitative data, thereby providing robust methodological support for an in-depth investigation of the differences in ceramide profiles among different dry skin subtypes. **Methods**: Stratum corneum samples were collected via tape stripping from 93 adult female volunteers, who were stratified into sensitive dry skin, non-sensitive dry skin, and normal skin groups based on clinical assessments. Cer metabolomics was analyzed via targeted metabolomics using liquid chromatography–tandem mass spectrometry (LC-MS/MS). **Results**: Quantitative analysis of ceramide content in different groups revealed significantly elevated levels of ultra-long-chain ceramides and the atypical Cer (d17:1/24:0) in the SD group, alongside relatively lower levels of shorter-chain ceramides. The NSD group, in contrast, was predominantly enriched in shorter-chain ceramides. Statistical analysis showed statistically significant differences in the levels of Cer (d18:1/24:0), Cer (d18:1/24:1), and Cer (d17:1/24:0) between the SD group and the N group. The UPLC-MS/MS method exhibits a wide linear range and high recovery. **Conclusions**: This method offers a reliable tool for the quantitative analysis of ceramides in dermatological, physiological, and pathological research. The findings not only underscore the profound heterogeneity in lipid metabolism underlying different dry skin subtypes but also provide a molecular rationale linking aberrant ceramide chain lengths to compromised barrier integrity and heightened inflammatory susceptibility. The partially validated analytical platform and the specific ceramide signatures revealed herein offer valuable tools and insights for advancing the mechanistic understanding, diagnosis, and targeted intervention of sensitive dry skin.

## 1. Introduction

The skin, as the body’s primary defense against external aggressors, relies critically on the integrity and function of its barrier. The stratum corneum (SC), the outermost layer of the epidermis, is central to this barrier function, being rich in various lipids. Among these, ceramides (Cers) are the most abundant intercellular lipids, constituting up to 50% of the stratum corneum lipids, and are pivotal for maintaining skin barrier function and regulating skin physiology [1]. Ceramides participate in multifaceted mechanisms, including acting as secondary messengers in signal transduction [2], serving as core intermediates in sphingomyelin metabolism, and forming specialized lamellar liquid crystal structures with cholesterol and free fatty acids (with a molar ratio of approximately 1:1:1) [3]. Crucially, ceramides exert diverse biological effects in preserving skin barrier function, notably by inhibiting transepidermal water loss (TEWL, reducing it by up to 30–50%) [4], enhancing corneocyte cohesion (increasing the mechanical stripping force required for SC removal by 2- to 3-fold) [5], and providing defense against the invasion of external pathogens [6].

Variations in ceramide content and composition are strongly linked to a range of skin concerns, notably sensitive dry skin. Studies have shown that individuals with dry skin often exhibit reduced total ceramide content and altered ceramide subclass profiles, which correlate with impaired barrier function and increased TEWL [7]. In sensitive skin—defined by amplified neurosensory responsiveness and reactive inflammatory activity—ceramide compositional shifts are even more pronounced, often marked by a substantial reduction in ultra-long-chain ceramides and a concomitant increase in short-chain or atypical ceramide species [8]. This imbalance between ultra-long-chain and short-chain ceramides appears to be a key factor in barrier dysfunction. Ultra-long-chain ceramides contribute to tightly packed, impermeable lipid layers, whereas shorter-chain ceramides enhance lipid fluidity and moisturization. In dry sensitive skin, this delicate balance is disrupted, leading to a compromised skin barrier with fragility and an increased predisposition to microcrack formation [9]. Recent lipidomics studies also highlight the role of specific ceramide classes in modulating skin sensitivity and hydration levels [10].

In terms of detection methodology, the analysis of ceramides has evolved significantly from traditional techniques to modern mass spectrometry-based approaches. Early analytical approaches including immunochemical assays, thin-layer chromatography (TLC), and high-performance liquid chromatography (HPLC) are plagued by inherent limitations such as insufficient specificity, low throughput, and the requirement for complex derivatization, which restricts their utility for comprehensive ceramide profiling [11,12,13,14,15]. In contrast, liquid chromatography–tandem mass spectrometry (LC–MS/MS) has emerged as the predominant platform due to its high sensitivity, selectivity, and ability to quantify multiple molecular species simultaneously without derivatization [16,17]. The use of electrospray ionization (ESI) coupled with multiple reaction monitoring (MRM) is particularly powerful for preserving molecular integrity and achieving precise quantification of low-abundance ceramides in complex biological matrices [18], solidifying the role of targeted LC-MS/MS as an indispensable tool for exploring lipid-mediated skin barrier dysfunction. Continued methodological optimization in recent years has further standardized high-throughput, quantitative panels for clinically relevant ceramides [19] and expanded the application of robust LC-MS/MS workflows to diverse biological samples, including those pertinent to dermatological research [20].

Leveraging the exceptional performance of ESI in handling thermally labile, highly polar, and high-molecular-weight compounds, and building upon previous untargeted lipidomics research findings that the composition of ceramides in sensitive dry skin has undergone significant changes [21], this study aimed to adopt an optimized LC-MS/MS platform to ensure the acquisition of reliable and accurate ceramide quantitative data, thereby providing robust methodological support for an in-depth investigation of the differences in ceramide profiles among different dry skin subtypes. Specifically, our method offers several key advantages over existing LC-MS/MS protocols: it achieves significantly lower limits of quantification (LOQs) compared to traditional methods (e.g., HPLC-UV assays often report LOQs of 50–100 ng/mL, while our method achieves LOQs as low as 0.008556 ng/mL), a rapid total chromatographic run time of only 7.5 min (compared to over 30 min for conventional methods), and a wide linear range of 0.5–50 ng/mL with high recovery rates (89.5% to 99.65% for high concentrations and 70% to 87% for the low concentration level). These enhancements represent a genuine methodological advancement, enabling more efficient and precise simultaneous quantification of multiple ceramide species. Utilizing this established methodology, we will deeply explore the characteristic changes in ceramide profiles among sensitive dry skin (SD), non-sensitive dry skin (NSD), and normal skin (N) groups. We anticipate that the present study will not only provide an effective and reliable tool for ceramide quantification in dermatological studies but, more importantly, elucidate the profound heterogeneity in lipid metabolism underlying distinct dry skin subtypes, thereby offering a molecular rationale linking aberrant ceramide chain lengths to compromised barrier integrity and heightened inflammatory susceptibility. Ultimately, this will provide valuable insights for advancing the mechanistic understanding, diagnosis, and targeted intervention strategies for sensitive dry skin.

## 2. Materials and Methods

### 2.1. Chemicals and Reagents

All ceramide reference standards, including Cer (d18:1/18:0) (purity ≥ 98%), Cer (d18:1/18:1) (≥98%), Cer (d18:1/20:0) (≥97%), Cer (d18:1/24:0) (≥98%), Cer (d18:1/24:1) (≥95%), and Cer (d17:1/24:0) (≥95%), were purchased from Sigma-Aldrich (St. Louis, MO, USA). Their molecular formulas and molecular weights are detailed in Table 1. These six ceramides were selected for targeted quantification based on our prior untargeted lipidomics data and their established biological relevance to skin barrier function, with Cer (d17:1/24:0) being of particular interest due to its less common C17-sphingoid base.

Deuterated internal standards, Cer (d18:1/18:0)-d7 (Cer18:0-d7, isotopic purity ≥ 98%), Cer (d18:1/24:0)-d7 (Cer24:0-d7, ≥98%), and Cer (d18:1/24:1)-d7 (Cer24:1-d7, ≥98%), were obtained from Alta Scientific Co., Ltd. (Tianjin, China). Therefore, internal standards were assigned to target ceramides based on structural similarity, particularly considering the acyl chain length, with the understanding that for certain analytes (e.g., Cer (d18:1/18:1), Cer (d18:1/20:0), and Cer (d17:1/24:0)), the use of structurally similar but non-isotopically matched internal standards may introduce potential ionization bias, extraction variability, and quantification inaccuracy. This strategic assignment aimed to minimize variability during sample preparation and LC-MS/MS analysis.

LC-MS grade solvents, including methanol (≥99.9%), isopropanol (≥99.8%), acetonitrile (≥99.9%), and formic acid (≥98%), were purchased from Thermo Fisher Scientific (Waltham, MA, USA). All other chemicals and reagents used were of analytical grade unless otherwise specified.

### 2.2. Chemical Reagents and Instruments

Filter paper disks with a diameter of 0.8 cm; pressure stick (225 g/cm^2^); sebum sampling tape (Sebutape®-S100, Clinical and Derm, Dallas, TX, USA); stratum corneum sampling tape (D-Squame®-D100, Clinical and Derm, Dallas, TX, USA); 4 mL Eppendorf (EP) tubes; analytical balance with a precision of 0.1 mg.

### 2.3. Instrumentation and Equipment

The LC-MS/MS analysis was performed using an ACQUITY UPLC I-Class Binary Solvent Manager (Waters, Milford, MA, USA) coupled with a Xevo TQ-S Triple Quadrupole Mass Spectrometer (Waters, Milford, MA, USA). Sample preparation utilized an MX-S Compact Digital Vortex Mixer (Scilogex, Rocky Hill, CT, USA) and a Nitrogen Evaporator (ANPEL, Shanghai, China).

### 2.4. Preparation of Standard and Internal Standard Solutions

Individual stock solutions of the six ceramide reference standards at high concentration were prepared by dissolving each standard in a methanol:isopropanol mixture (1:1, *v*/*v*) to a final concentration of 500 μg/mL. Aliquots of these individual stock solutions were then combined and diluted with methanol to prepare a mixed standard stock solution in which each of the six ceramide reference standards was at a concentration of 100 ng/mL.

Similarly, Individual stock solutions of the three deuterated internal standards at high concentration were prepared by dissolving each standard in methanol to 500 μg/mL. These were then combined and diluted with methanol to yield a mixed internal standard stock solution at 100 ng/mL.

### 2.5. Processing of Standard Solutions

The 100 ng/mL mixed standard stock solution was serially diluted with methanol to prepare a calibration curve consisting of seven concentration points: 50, 20, 10, 5, 2, 1, and 0.5 ng/mL. A fixed volume (250 μL) of the 100 ng/mL mixed internal standard stock solution was added to each calibration level. The calibration solutions, covering a concentration range of 0.5–50 ng/mL for the six target ceramides (Cer18:0, Cer18:1, Cer20:0, Cer24:0, Cer24:1, and Cer d17:1/24:0), were analyzed. Each concentration level was injected in duplicate. A linear regression equation was established from the plotted calibration curve, and the coefficient of determination (R^2^) was calculated to assess linearity.

### 2.6. Study Subject Grouping and Sample Collection

#### 2.6.1. Study Participants

Participants in this study were recruited from Zhengzhou University and classified into three groups: sensitive dry skin (SD), non-sensitive dry skin (NSD), and normal skin (N). Grouping was based on objective, instrument-measured parameters and a standardized clinical test. The inclusion criteria were as follows: (1) female college students aged between 18 and 25 years old; (2) SD group: individuals demonstrating positive lactic acid tingling, a Corneometer value of 45 a.u. or below at the aforementioned intersection, and an oil content below 120 μg/cm^2^; (3) NSD group: individuals with negative lactic acid tingling responses, a Corneometer value of 45 a.u. or below at the aforementioned intersection, and a forehead skin sebum content of less than 120 μg/cm^2^; (4) N group: individuals without positive lactic acid tingling, an oil content below 120 μg/cm^2^, and a Corneometer value exceeding 45 a.u. at the aforementioned intersection. The exclusion criteria were as follows: (1) subjects who were pregnant, breastfeeding, or planning to become pregnant; and those (2) suffering from severe systemic diseases. A total of 93 study subjects were finally enrolled, including 32 SD, 29 NSD, and 32 N. This study was approved by the Life Sciences Ethics Review Committee of Zhengzhou University. Before the experiment began, all volunteers were informed of the purpose of the experiment and signed an informed consent form.

#### 2.6.2. Test Environmental Conditions

Throughout the instrumental testing session, the test should be controlled in an environment with a temperature of 20 ± 2 °C, a relative humidity of 50 ± 10% RH, and a color temperature of 6500 K.

#### 2.6.3. Physiological Indicator Measurements

Volunteers were required to clean their faces with water at 21:00 on the previous day, after which re-cleansing or the use of any skincare/makeup products was prohibited until the start of the experiment. Upon arrival, the subjects first cleansed their faces using water. They were then placed in an environment with constant temperature and humidity for 30 min to stabilize their skin condition. Then, Sebumeter, Corneometer, and Tewameter were used to measure the stratum corneum water content, oil content, and TEWL, respectively, and the data obtained were analyzed and recorded.

#### 2.6.4. Skin Lipid Sampling

After completing the physiological index measurements, the lipid peeling tape method was employed to collect sebaceous gland lipids and stratum corneum intercellular lipids from the volunteers’ faces. (1) Initially, the grease tape was attached to the sebaceous gland lipid sampling site, and the pressure bar was utilized to press evenly for 3 s to ensure that the tape was tightly adhered to the skin. After 30 min, the tape was carefully removed and placed in a 4 mL EP tube. (2) After the sebaceous gland lipid sampling, the stratum corneum intercellular lipids were sampled. The stratum corneum tape was applied to the same sampling site, pressed with a pressure bar for 15 s, quickly removed, and placed into the corresponding 4 mL EP tube. (3) The tape and the EP tube were weighed accurately after sampling, and the samples were stored in a refrigerator at −80 °C immediately after completion for subsequent analysis.

### 2.7. Sample Preparation

The sample preparation commenced by retrieving the stored skin tape strips from the −80 °C freezer. Subsequently, 2 mL of methanol and 250 μL of the 100 ng/mL internal standard stock solution were added to the Eppendorf tubes containing the tape samples. The mixtures were vortexed for 1 min, followed by incubation with shaking at room temperature for 1 h to ensure efficient lipid extraction. After incubation, the sampling tapes were carefully removed from the sample tubes, and the resulting extracts were retained. Under controlled room temperature (25 ± 2 °C), the extracts were evaporated to complete dryness using a nitrogen evaporator with a gas flow rate set at approximately 15 L/min and high-purity nitrogen gas (>99.9999%). The dried residues were then stored at −20 °C until further analysis. Before LC-MS/MS analysis, the dried samples were reconstituted in 1 mL of a solvent mixture comprising acetonitrile, isopropanol, and water (65:30:5, *v*/*v*/*v*), followed by vortexing for 30 s. The resulting solution was syringe-filtered through a 0.22 μm PTFE membrane and transferred to autosampler vials for analysis.

### 2.8. Quantification of Ceramide Content

Calculation Methodology:

W = CV/S

where

W: Ceramide content (μg/cm^2^);

C: Mass concentration of the ceramide (ng/mL);

V: Volume of the sample solution (μL);

S: Area of the tape sample (3.8 cm^2^).

### 2.9. LC-MS/MS Analytical Conditions

The LC-MS/MS system was operated under optimized conditions to ensure high sensitivity and selectivity.

#### 2.9.1. MRM Transition and Compound-Specific Instrument Parameter Optimization

The standard solutions were directly infused into the mass spectrometer to systematically optimize the precursor ions, product ions, cone voltage, and collision energy. Product ion scan and parameter optimization were performed separately for native ceramides and d7-labeled internal standards using the built-in MRM auto-optimization software of the instrument. Analysis was performed using electrospray ionization (ESI) in positive ion mode with multiple reaction monitoring (MRM). The detailed mass spectrometric conditions are summarized in Table 2.

#### 2.9.2. Electrospray Ionization Source and Interface Parameters

Based on preliminary chromatographic and mass spectrometric conditions, the mobile phase composition and gradient elution program were systematically optimized by evaluating the actual peak characteristics, retention behavior, and signal response of the analytes. This optimization aimed to achieve satisfactory chromatographic resolution, ensuring distinct separation of different molecular species without interference, along with peaks exhibiting acceptable shape and intensity. The corresponding mass spectrometer parameters are provided in Table 3.

#### 2.9.3. Liquid Chromatography Method

The chromatographic separation was performed on an ACQUITY UPLC BEH C18 column (2.1 mm × 50 mm, 1.7 μm, Waters Corporation, Milford, MA, USA) maintained at 40 °C. The mobile phase consisted of (A) 0.1% formic acid in water and (B) 0.1% formic acid in isopropanol/acetonitrile (1:1, *v*/*v*). The flow rate was set at 0.3 mL/min with an injection volume of 5 μL. A gradient elution program was employed, as detailed in Table 4.

### 2.10. Method Validation

#### 2.10.1. Calibration Curve and Its Linear Concentration Range

A series of seven standard working solutions at varying concentrations (0.5–50 ng/mL) were prepared using the reference standards and internal standards. To monitor system variability, all standard solutions were injected in duplicate. The construction of the calibration curve was formally defined by a bivariate coordinate system wherein the abscissa (*X*-axis) represented the precisely known nominal concentration of the analyte in the series of prepared standard solutions, and the ordinate (*Y*-axis) referred to the ratio of the analyte signal to the internal standard signal. The accuracy at each concentration level, expressed as the percentage deviation from the nominal value, should be within ±20%, and no fewer than 80% of the concentration levels must meet this acceptance criterion.

#### 2.10.2. Limit of Detection and Limit of Quantification

Blank tape samples were processed and analyzed using the identical procedures and reagents employed for the actual skin tape samples. Each analytical batch included these procedural blanks for quality control. When abnormal blank values were detected, a root cause investigation was initiated. A dedicated log was established for the consistent monitoring of blank values during analysis.

The limit of detection (LOD) is defined as the lowest concentration of an analyte that the instrument can reliably detect, even if the instrument cannot necessarily quantify this concentration with adequate accuracy and precision. The limit of quantification (LOQ) refers to the lowest concentration that can be determined with satisfactory accuracy, precision, and reliability under the stated methodological conditions. These limits were calculated based on the response variability obtained from repeated measurements of blank samples and the slope of the established calibration curve, providing specific numerical estimates for the lowest detectable and quantifiable concentrations.

LOD and LOQ in ng/mL are calculated as follows:

LOD = 3.3σ/S

LOQ = 10σ/S

where

σ: the standard deviation of the response;

S: the slope of the calibration curve.

#### 2.10.3. Spike Recovery

We evaluated the method’s accuracy via spike-and-recovery experiments. Blank tape samples were spiked with standard solutions at low and high concentration levels, with three replicate determinations per level. The recovery rate was then calculated for each concentration.

The formula for calculating the spike recovery rate (R%) is as follows:

R% = (c_m_ − c_o_)/c_s_ × 100%

where

R%: Recovery rate;

c_m_: Measured concentration of the spiked sample;

c_o_: Original concentration of the unspiked sample (background);

c_s_: Nominal concentration of the spike (amount added).

However, detailed evaluations of intra-day and inter-day precision, carry-over effects, and analyte stability under various conditions were not performed in this study.

### 2.11. Statistical Method

Data are expressed as mean ± standard deviation. Prior to statistical analysis, the normality of the data distribution was assessed using the Shapiro–Wilk test, and the homogeneity of variance was verified via Levene’s test. No formal outlier analysis was performed prior to statistical comparisons, a limitation acknowledged due to the exploratory nature of this study. For each of the six ceramides, multiple group comparisons of their levels among the sensitive dry skin, non-sensitive dry skin and normal skin groups were performed using one-way analysis of variance (ANOVA), followed by Tukey’s post hoc test for pairwise comparisons. Bonferroni correction was applied for multiple testing to control the type I error in the pairwise comparisons across the three groups for each ceramide. A value of *p* < 0.05 was considered statistically significant. All statistical analyses were conducted using SPSS version 25.0 (IBM Corp., Armonk, NY, USA).

## 3. Results

### 3.1. Standard Curve Experimental Results

All six target ceramides demonstrated excellent linearity over the concentration range of 0.5–50 ng/mL, with correlation coefficients (R^2^) greater than 0.99 (Table 5). This established linear range and sensitivity markedly outperform those typically achieved by traditional methods. For instance, conventional assays for ceramides often report limits of quantification (LOQ) in the range of 50–100 ng/mL and require lengthy run times exceeding 30 min per sample [13,14]. In contrast, the present LC-MS/MS method achieved significantly lower LOQs and a total chromatographic run time of only 7.5 min. This combination of ultra-high sensitivity, wide dynamic range, and rapid analysis time underscores a substantial improvement in throughput and precision for the simultaneous quantification of multiple ceramide species, effectively addressing the limitations of prior methodologies.

### 3.2. Limits of Detection and Quantification

The limits of detection (LOD) and quantification (LOQ) for the six ceramides were calculated based on multiple blank measurements and the established calibration curve. The results are shown in Table 6.

### 3.3. Spike Recovery Results

Blank tape samples were spiked with ceramide standard solutions at concentrations of 1 ng/mL (low) and 20 ng/mL (high), and processed following the aforementioned sample preparation and instrumental methodology. Each concentration level was prepared in triplicate (*n* = 3). The observed spike recovery rates ranged from 89.5% to 99.65% for the high concentration level and from 70% to 87% for the low concentration level. These results demonstrate that the method provides acceptable accuracy for quantitative analysis, with the high concentration level exhibiting excellent recovery performance.

The corresponding spike recovery rates at all tested concentration levels are summarized in Table 7.

### 3.4. Quantification of Skin Ceramides in Different Population Groups

**Figure 1:**
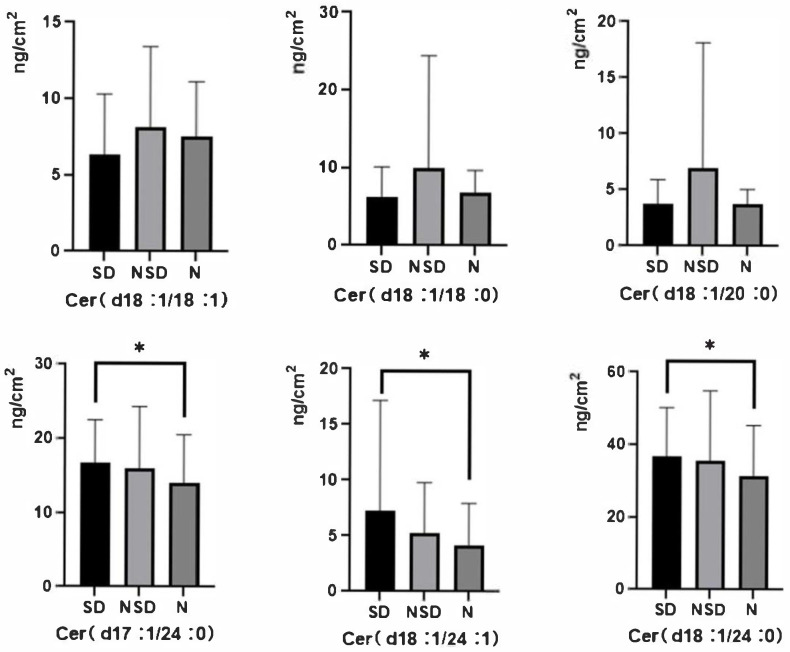
Ceramide Content in Sensitive Dry Skin, Non-Sensitive Dry Skin, and Normal Skin Groups. The horizontal axis represents the different groups, and the vertical axis denotes the ceramide content in the corresponding samples (mean ± SD). * indicates *p* < 0.05 (statistically significant).

The measured responses from the skin lipid tape sample solutions were converted to mass concentrations (ng/cm^2^) of ceramides using the respective linear regression equations derived from the calibration curves. Subsequently, the content of each of the six target ceramides was statistically summarized for each study group, with the results expressed as mean ± standard deviation (mean ± SD), as detailed in Table 8 and Table 9, and Figure 1.

Quantitative analysis revealed statistically significant differences in the composition and levels of ceramides in the stratum corneum lipids among the three population groups. Specifically, the NSD group exhibited relatively higher levels of Cer (d18:1/18:0) (10.0 ± 14.4 ng/cm^2^), Cer (d18:1/18:1) (8.1 ± 5.3 ng/cm^2^), and Cer (d18:1/20:0) (6.9 ± 11.2 ng/cm^2^) compared to the SD skin and N groups. In contrast, the levels of Cer (d18:1/24:0), Cer (d18:1/24:1), and Cer (d17:1/24:0) in the NSD group were lower than those in the SD skin group, but remained elevated relative to the N group.

In contrast, the SD group demonstrated significantly elevated levels of Cer (d18:1/24:0) (36.8 ± 13.3 ng/cm^2^), Cer (d18:1/24:1) (7.2 ± 9.9 ng/cm^2^), and Cer (d17:1/24:0) (16.7 ± 5.8 ng/cm^2^). At the same time, the concentrations of Cer (d18:1/18:0), Cer (d18:1/18:1), and Cer (d18:1/20:0) were relatively lower compared to the other two groups.

Statistical analysis revealed that the levels of Cer (d18:1/24:0), Cer (d18:1/24:1), and Cer (d17:1/24:0) showed statistically significant differences (*p* < 0.05) between the SD group and the N group.

## 4. Discussion

Liquid chromatography–tandem mass spectrometry (LC-MS/MS), as a powerful analytical technique emerging in metabolomics, has garnered increasing attention and widespread application. It is recognized as the “gold standard” for quantifying endogenous small molecule metabolites, including ceramides [22]. Ceramides have been identified as potential biomarkers in various disease studies, holding significant promise for predicting disease progression and prognosis. This study developed and partially validated a quantitative LC-MS/MS method for the simultaneous determination of multiple ceramide species. Through systematic optimization and exploratory validation of instrumental conditions, mass spectrometric parameters, and chromatographic settings, we established an effective and reliable platform for ceramide detection. This methodology provides robust technical support for comprehensive ceramide analysis in biological samples and holds the potential to expand its application across various diseases and clinical scenarios.

The results indicate that the non-sensitive dry skin (NSD) group was predominantly enriched in shorter-chain ceramides (e.g., Cer (d18:1/18:0), Cer (d18:1/18:1), and Cer (d18:1/20:0)), whereas the sensitive dry skin (SD) group exhibited a marked elevation of ultra-long-chain ceramides (e.g., Cer (d18:1/24:0), Cer (d18:1/24:1), and the atypical Cer (d17:1/24:0)). This distinct ceramide chain length profile may suggest a difference in the mechanisms underlying barrier dysfunction between these two dry skin subtypes. Notably, the elevated ultra-long-chain ceramides in the SD group observed herein contrasts with previous reports, which described a substantial reduction in ultra-long-chain ceramide species in sensitive or barrier-impaired skin. As highlighted by Fujii [8], sensitive skin (characterized by amplified neurosensory responsiveness and reactive inflammatory activity) typically exhibits ceramide compositional shifts marked by diminished ultra-long-chain ceramides and concurrent increases in short-chain or atypical species. This apparent discrepancy is a significant finding that warrants careful consideration, as it potentially highlights the heterogeneity of sensitive skin phenotypes. Our observation of elevated ultra-long-chain ceramides in the SD group, while contrasting with some prior literature, is presented cautiously and interpreted as hypothesis-generating, suggesting that this finding might be specific to our study population, the precise phenotypic definition of “sensitive skin” employed, or the severity of the barrier impairment in our cohort, which may differ from conditions like overt chronic inflammatory skin diseases (e.g., severe atopic dermatitis or chronic irritant contact dermatitis) where sustained immune activation disrupts lipid biosynthesis enzymes such as ELOVL1 and ELOVL4. In this specific phenotype, the skin barrier may initiate a putative maladaptive compensatory upregulation of ultra-long-chain ceramides in an attempt to reinforce physical barrier rigidity in response to dryness-induced mild barrier damage. However, this excessive, unregulated elevation fails to form the ordered lamellar structures typical of healthy skin—likely due to the lack of coordinated synthesis of accompanying lipids (e.g., cholesterol and free fatty acids) [8]—and instead could lead to pathological lipid aggregation.

In sensitive dry skin, the significantly elevated levels of ultra-long-chain ceramides—exemplified by Cer (d18:1/24:0)—disrupt the homeostatic balance of cutaneous ceramide composition [23]. Excessive aggregation of ultra-long-chain ceramides may lead to the formation of an unduly compact lipid architecture, characterized by substantially diminished flexibility and a resemblance to a rigid, plate-like structure. This hyper-rigid stratum corneum is highly susceptible to microcrack formation upon exposure to minor external pressure or friction. The development of such microcracks could compromise skin barrier integrity, potentially creating micro-pathways for the penetration of external irritants. Once these irritants infiltrate the skin, they are recognized by the immune system, triggering immune activation and inflammatory responses. The subsequent release of various inflammatory mediators, such as histamine and interleukins, induces a range of sensitive skin symptoms, including erythema, pruritus, and stinging sensations [24]. Moreover, the inflammatory response further compromises the structural and functional integrity of the cutaneous barrier, establishing a vicious cycle that perpetuates and exacerbates barrier compromise [25]. This maladaptive compensatory mechanism might explain the unique ultra-long-chain ceramide profile in the sensitive dry skin subtype investigated herein, highlighting the phenotypic specificity of ceramide metabolic dysregulation in different forms of sensitive/barrier-impaired skin. It is plausible that if the SD subtype progresses to chronic inflammation, the initial elevation of ultra-long-chain ceramides may transition to the reduction, reflecting a shift from compensatory to degenerative lipid metabolism.

Beyond chain length variation, structural heterogeneity among ceramides warrants attention. The presence of the atypical ceramide species Cer (d17:1/24:0) in sensitive dry skin suggests a potential dysregulation in the sphingoid base metabolic pathway. This ceramide is characterized by a sphingoid base composed of 17 carbon atoms with one double bond (d17:1), which is structurally distinct from the canonical ceramides in healthy skin that are predominantly composed of 18-carbon sphingoid bases (e.g., d18:1) [26]. Aberrant upregulation of SPTLC2 (serine palmitoyltransferase long-chain subunit 2), a key enzyme in the sphingoid base biosynthesis pathway, may serve as a critical trigger for the generation of such atypical ceramide species. As the central regulatory component in de novo sphingosine synthesis, altered SPTLC2 activity directly modulates the structural repertoire of sphingoid bases, consequently reshaping the molecular diversity of downstream ceramide profiles [27,28]. Notably, Fujii [8] also reported increased atypical ceramide species in sensitive/barrier-impaired skin, which aligns with the present findings—suggesting that while ultra-long-chain ceramide quantity differs between studies, structural dysregulation of ceramides may be a common feature of sensitive skin phenotypes. The emergence of such atypical ceramides, in conjunction with the maladaptive elevation of ultra-long-chain ceramides, may be associated with the pathological processes of sensitive dry skin via distinct molecular mechanisms, thereby offering novel insights into the pathogenesis of this specific skin phenotype.

While this study provides valuable insights and generates novel testable hypotheses regarding ceramide-mediated mechanisms in sensitive dry skin, several limitations should be acknowledged. Firstly, the relatively small sample size may have contributed to the observed high standard deviations, potentially compromising the statistical power and generalizability of the findings. Future studies with expanded cohorts are necessary to validate the hypotheses generated herein, particularly by comparing ceramide profiles across different sensitive skin phenotypes (e.g., inflammatory vs. non-inflammatory subtypes) to confirm the specificity of ultra-long-chain ceramide elevation. Secondly, the absence of transcriptomic or enzymatic activity data limits our ability to elucidate the molecular mechanisms underlying the ceramide alterations. Integrating multi-omics approaches—such as transcriptomics, proteomics, and metabolomics—in future work could uncover the regulatory networks governing ceramide metabolism, thereby providing a more robust theoretical foundation for the treatment and prevention of sensitive dry skin. Further research should focus on verifying the regulatory effect of specific ceramides on barrier function through intervention experiments, such as using ceramide analogues or inhibitors to observe changes in skin barrier function; and developing lipid supplementation strategies for sensitive dry skin, such as regulating the ratio of ultra-long-chain to short-chain ceramides to improve skin barrier function and alleviate symptoms of skin sensitivity and dryness. Additionally, future studies should explore the temporal dynamics of ceramide alterations in sensitive dry skin to determine whether the observed elevation of ultra-long-chain ceramides is a transient compensatory response or a stable phenotypic feature. Furthermore, it is important to acknowledge that all participants in this study were adult females aged between 18 and 25 years old, recruited from a single center. This demographic specificity, coupled with the single-center nature of the study, significantly limits the generalizability of our findings to other populations and contexts. Consequently, the conclusions drawn from this study are primarily applicable to this specific cohort and should be interpreted with caution when considering broader populations. While we did not specifically control for individual hormonal status, cosmetic usage, or a comprehensive range of environmental factors, participants were recruited from a relatively homogeneous university population, and clinical assessments were performed under standardized environmental conditions (temperature of 20 ± 2 °C, relative humidity of 50 ± 10% RH). The exclusion of male participants was primarily due to the observed higher prevalence of sensitive skin conditions in females within our target demographic, as well as practical considerations for standardizing the study population. Future research should include a more diverse cohort, encompassing different age groups, genders, and ethnicities, and systematically evaluate the influence of hormonal fluctuations, cosmetic regimens, and varied environmental exposures on ceramide profiles in sensitive dry skin.

Due to the unavailability of specific isotopically labeled internal standards for some target ceramide species (e.g., Cer (d18:1/18:1), Cer (d18:1/20:0), and Cer (d17:1/24:0)), structurally similar deuterated ceramides were used as surrogate internal standards. This approach, while necessary, means these surrogate internal standards may not fully compensate for potential differences in extraction efficiency and matrix effects compared to their respective unlabeled counterparts, which could introduce a degree of quantitative uncertainty for these specific ceramides. This limitation should be considered when interpreting the quantitative data for Cer (d18:1/18:1), Cer (d18:1/20:0), and Cer (d17:1/24:0), and claims regarding their absolute quantitative accuracy are tempered accordingly. Future efforts will aim to synthesize or acquire a broader range of isotopically labeled internal standards to enhance the accuracy and precision of ceramide quantification across all species.

Additionally, high variability was observed for certain ceramides, particularly in the NSD group. This substantial standard deviation may stem from individual differences in skin physiology, lifestyle, or unmeasured environmental factors. It is also possible that the analytical reproducibility, despite our efforts in method optimization, might have contributed to this variability, especially for analytes present at lower concentrations or those with more complex matrix interactions. Future studies with larger sample sizes and more stringent stratification criteria, coupled with enhanced analytical precision, will be crucial to further delineate the true biological variations and minimize measurement noise. Consequently, the statistical robustness of our findings, particularly for non-significant results, should be interpreted with caution, acknowledging the potential for Type II errors due to the modest sample size and observed data variability.

Finally, we recognize that while our LC-MS/MS method demonstrated excellent linearity, high sensitivity (low LOD/LOQ values), and acceptable spike recovery rates, detailed evaluations of intra-day and inter-day precision, carry-over, and the analyte stability under various conditions were not performed. These parameters account for potential variability and interferences inherent in complex biological matrices. This represents a limitation of the current work, primarily due to the initial scope and resource constraints of the study. However, the consistent performance observed during routine sample analysis, coupled with the rigorous optimization of chromatographic and mass spectrometric conditions, provides confidence in the qualitative and quantitative trends reported. Future studies will prioritize a more exhaustive method validation, incorporating these essential parameters to further strengthen the analytical platform and ensure the highest level of data integrity and reproducibility.

## 5. Conclusions

In summary, this study successfully established and partially validated a sensitive, high-throughput LC-MS/MS method for the targeted quantification of six ceramide species. By applying this methodology, we identified a distinct ceramide profile in sensitive dry skin, characterized by a significant elevation of ultra-long-chain species (Cer (d18:1/24:0), Cer (d18:1/24:1)) and the atypical Cer (d17:1/24:0), alongside a relative depletion of shorter-chain ceramides. These findings not only underscore the profound heterogeneity in lipid metabolism underlying distinct dry skin subtypes but also furnish a molecular rationale elucidating the association between aberrant ceramide chain lengths and compromised barrier integrity, as well as heightened inflammatory susceptibility. The validated analytical platform and the specific ceramide signatures revealed herein offer valuable tools and insights for advancing the mechanistic understanding, diagnosis, and targeted intervention of sensitive dry skin.

## Figures and Tables

**Table 1:** Ceramide Reference Standards: Chemical Names, Molecular Formulas, and Molecular Weights.

Name	Molecular Formula	Molecular Weight
Cer (d18:1/18:0)	C_36_H_71_NO_3_	565.95
Cer (d18:1/18:1)	C_36_H_69_NO_3_	563.94
Cer (d18:1/20:0)	C_38_H_75_NO_3_	594.01
Cer (d18:1/24:0)	C_42_H_83_NO_3_	650.11
Cer (d18:1/24:1)	C_42_H_81_NO_3_	648.10
Cer (d17:1/24:0)	C_41_H_81_NO_3_	636.08

**Table 2:** Mass Spectrometric Parameters for Quantitative Analysis of Ceramides in Multiple Reaction Monitoring (MRM) Mode.

Chemical Name	Precursor Lon (*m*/*z*)	Product Lon (*m*/*z*)	Cone Voltage	Collision Energy (V)
Cer (d18:1/18:0)	566.41	264.26	10	34
Cer (d18:1/18:1)	564.53	264.26	40	22
Cer (d18:1/20:0)	594.00	264.00	30	30
Cer (d18:1/24:0)	650.57	264.25	34	34
Cer (d18:1/24:1)	648.10	264.25	2	28
Cer (d17:1/24:0)	636.73	250.27	30	32
Cer (d18:1/18:0)-d7	573.21	271.21	50	30
Cer (d18:1/24:0)-d7	657.11	271.26	26	26
Cer (d18:1/24:1)-d7	655.65	271.28	34	32

**Table 3:** Mass Spectrometer Parameters.

Name	Parameters
Capillary Voltage	3.0 kv
Cone Voltage	35 v
Source Temperature	150 °C
Desolvation Temperature	800 L/h
Cone Gas Flow	50 L/h
Curtain Gas Flow	25 psi
Ion Spray Voltage	5000 v
Temperature	300 °C
Gas 1 (Nebulizer Gas)	50 psi
Gas 2 (Heater Gas)	30 psi
Declustering Potential	30 v
Entrance Potential	10 v
Cell Exit Potential	20 v
Collision Energy	40 ev

**Table 4:** Gradient Program of the Mobile Phase.

Gradient Program	Time (min)	Flow Rate (mL/min)	Mobile Phase A (%)	Mobile Phase B (%)
1	0	0.3	15	85
2	0.3	0.3	1	99
3	5.5	0.3	1	99
4	5.51	0.3	15	85
5	7.5	0.3	15	85

**Table 5:** Calibration Curve Results for Ceramides.

Name	Calibration Equation	R^2^	RT	Linear Range (ng/mL)	Internal Standard
Cer (d18:1/18:0)	2.88426·× − 0.494713	0.997281	3.47	0.5–50	Cer (d18:1/18:0)-d7
Cer (d18:1/18:1)	0.876969·× − 0.0902798	0.994294	3.11	0.5–50	Cer (d18:1/18:0)-d7
Cer (d18:1/20:0)	2.36207·× − 0.875852	0.995026	3.83	0.5–50	Cer (d18:1/18:0)-d7
Cer (d18:1/24:0)	20.824·× − 4.82869	0.997779	4.66	0.5–50	Cer (d18:1/24:0)-d7
Cer (d18:1/24:1)	0.98943·× − 0.265936	0.99701	4.08	0.5–50	Cer (d18:1/24:1)-d7
Cer (d17:1/24:0)	23.9543·× − 6.31083	0.995434	4.42	0.5–50	Cer (d18:1/24:0)-d7

**Table 6:** Limits of Detection and Quantification (ng/mL) for Individual Ceramides.

	Cer (d18:1/18:0)	Cer (d18:1/18:1)	Cer (d18:1/20:0)	Cer (d18:1/24:0)	Cer (d18:1/24:1)	Cer (d17:1/24:0)
σ	0.04077	0.009741	0.006976	0.00947	0.09434	0.06211
S	2.88426	0.876969	2.36207	0.98943	20.824	23.9543
LOD	0.04665	0.03666	0.009746	0.03158	0.01495	0.008556
LOQ	0.14136	0.11108	0.02953	0.09571	0.0453	0.02593

**Table 7:** Spike Recovery Rates at Different Concentration Levels.

Name	C_spiked_	C_measured_	C_background_	Average Recovery (%)
Cer (d18:1/18:0)	1	0.8	0.2	77
		1.1	0.2	
		1.0	0.2	
	20	20.4	0.2	89.5
		14.5	0.2	
		19.4	0.2	
Cer (d18:1/18:1)	1	0.7	0.2	70
		1.0	0.2	
		1.0	0.2	
	20	14.1	0.2	94
		15.4	0.2	
		26.9	0.2	
Cer (d18:1/20:0)	1	0.6	0.2	70
		1.1	0.2	
		1.0	0.2	
	20	22.7	0.2	99.65
		14.4	0.2	
		22.7	0.2	
Cer (d18:1/24:0)	1	0.7	0.3	77
		1.5	0.3	
		1.0	0.3	
	20	18.1	0.3	95.65
		20.5	0.3	
		19.7	0.3	
Cer (d18:1/24:1)	1	0.8	0.3	70
		1.1	0.3	
		1.1	0.3	
	20	20.2	0.3	92
		14.3	0.3	
		21.5	0.3	
Cer (d17:1/24:0)	1	0.8	0.3	87
		1.1	0.3	
		1.6	0.3	
	20	20.9	0.3	97.15
		18.4	0.3	
		19.9	0.3	

**Table 8:** Content of the Six Ceramides in Stratum Corneum Lipids Across Study Groups (ng/cm^2^).

	SD	NSD	N
Cer (d18:1/18:0)	6.3 ± 3.8	10.0 ± 14.4	6.9 ± 2.8
Cer (d18:1/18:1)	6.3 ± 4.0	8.1 ± 5.3	7.5 ± 3.6
Cer (d18:1/20:0)	3.7 ± 2.2	6.9 ± 11.2	3.7 ± 1.3
Cer (d18:1/24:0)	36.8 ± 13.3	35.5 ± 19.3	31.3 ± 14.0
Cer (d18:1/24:1)	7.2 ± 9.9	5.2 ± 4.6	4.2 ± 3.8
Cer (d17:1/24:0)	16.7 ± 5.8	16.0 ± 8.3	14.0 ± 6.6

**Table 9:** Statistical Differences in Six Ceramide Levels in Stratum Corneum Lipids Across Study Groups.

	SD vs. N	NSD vs. N	SD vs. NSD
Cer (d18:1/18:0)	0.256	0.727	0.300
Cer (d18:1/18:1)	0.216	0.975	0.153
Cer (d18:1/20:0)	0.582	0.144	0.083
Cer (d18:1/24:0)	0.027 *	0.499	0.192
Cer (d18:1/24:1)	0.043 *	0.413	0.329
Cer (d17:1/24:0)	0.015 *	0.302	0.210

* *p* < 0.05 (statistically significant).

## Data Availability

The raw data supporting the conclusions of this article will be made available by the authors on request.

## Abbreviations

The following abbreviations are used in this manuscript:

LC-MS/MS	liquid chromatography–tandem mass spectrometry
SD	dry skin
NSD	non-sensitive dry skin
N	normal skin
SC	stratum corneum
Cers	ceramides
TEWL	transepidermal water loss
TLC	thin-layer chromatography
HPLC	high-performance liquid chromatography
ESI	electrospray ionization
MRM	multiple reaction monitoring
UPLC-MS/MS	ultra-performance liquid chromatography–tandem mass spectrometry
LOD	limit of detection
LOQ	limit of quantification
ANOVA	one-way analysis of variance
SPTLC2	serine palmitoyltransferase long-chain subunit 2
